# Systemic inflammatory response predicts outcome in patients undergoing resection for ductal adenocarcinoma head of pancreas

**DOI:** 10.1038/sj.bjc.6602305

**Published:** 2004-12-14

**Authors:** N B Jamieson, P Glen, D C McMillan, C J McKay, A K Foulis, R Carter, C W Imrie

**Affiliations:** 1 University Department of Surgery, Royal Infirmary, Glasgow G31 2ER, UK; 2 Department of Pathology, Royal Infirmary, Glasgow G31 2ER, UK

**Keywords:** pancreatic cancer, curative resection, tumour size, C-reactive protein, survival

## Abstract

The aim of the present study was to examine the relationship between the clinicopathological status, the pre- and postoperative systemic inflammatory response and survival in patients undergoing potentially curative resection for ductal adenocarcinoma of the head of the pancreas. Patients (*n*=65) who underwent resection of ductal adenocarcinoma of the head of pancreas between 1993 and 2001, and had pre- and postoperative measurements of C-reactive protein, were included in the study. The majority of patients had stage III disease (International Union Against Cancer Criteria, IUCC), positive circumferential margin involvement (R_1_), tumour size greater than 25 mm with perineural and lymph node invasion and died within the follow-up period. On multivariate analysis, tumour size (hazard ratio (HR) 2.10, 95% confidence interval (CI) 1.20–3.68, *P*=0.009), vascular invasion (HR 2.58, 95% CI 1.48–4.50, *P*<0.001) and postoperative C-reactive protein (HR 2.00, 95% CI 1.14–3.52, *P*=0.015) retained independent significance. Those patients with a postoperative C-reactive protein ⩽10 mg l^−1^ had a median survival of 21.5 months compared with 8.4 months in those patients with a C-reactive protein >10 mg l^−1^ (*P*<0.001). The results of the present study indicate that, in patients who have undergone potentially curative resection for ductal adenocarcinoma of the head of pancreas, the presence of a systemic inflammatory response predicts poor outcome.

The outlook for patients with ductal adenocarcinoma of the head of the pancreas remains poor, having the lowest 5-year survival rate of any cancer (Parker *et al*, 1996). Surgery remains the only proven approach for improving survival in these patients. However, surgery is complicated and is associated with appreciable morbidity and mortality. As a consequence, potentially curative surgery is carried out relatively infrequently and usually in a specialist centre.

The prognosis for patients who undergo potentially curative resection has been reported to be determined by various pathologic characteristics of the resected tumour specimen. Pathologic predictors of survival after surgery include vascular invasion (Griffanti-Bartoli *et al*, 1994), perineural invasion (Hermanek, 1998; Ozaki *et al*, 1999), histological tumour grade (Greer and Brennan, 1993), not achieving a clear margin (Yeo *et al*, 1995) and tumour size (Fortner *et al*, 1996). Taking all these factors into account, Fortner and co-workers (1996) reported that, in 52 patients undergoing potentially curative regional pancreatectomy, tumour size was the strongest predictor of survival independent of lymph node metastases.

It is increasingly recognised that it is not only the intrinsic properties of tumour cells which determine tumour spread but also the host inflammatory response (Balkwill and Mantovani, 2001; Coussens and Werb, 2002). Indeed, the systemic inflammatory response, as evidenced by elevated circulating concentrations of C-reactive protein, has been shown to be a disease-independent prognostic factor in a variety of operable tumours (Mahmoud and Rivera, 2002; McMillan *et al*, 2003; Ikeda *et al*, 2003). In particular, an elevated C-reactive protein, measured either prior to or following curative surgery, has been shown to predict recurrence and overall survival, independent of stage, in patients with colorectal cancer (McMillan *et al*, 2003).

An elevated C-reactive protein concentration has previously been shown to have independent prognostic value in patients with unresectable pancreatic ductal adenocarcinoma (Falconer *et al*, 1995; Ueno *et al*, 2000; Engelken *et al*, 2003). However, to our knowledge the prognostic value of C-reactive protein has not been previously examined in patients with operable disease.

The aim of the present study was to examine the relationship between clinicopathologic status, the systemic inflammatory response and survival in patients undergoing potentially curative resection for ductal adenocarcinoma of the head of the pancreas.

## PATIENTS AND METHODS

### Patients

Patients who on the basis of radiological and pathological staging underwent resection of ductal adenocarcinoma of the head of pancreas (between 1st January 1993 and 31st July 2001) had pre- and postoperative measurements of C-reactive protein, and postoperative measurements of tumour size were included in the study. Measurement of C-reactive protein was carried out on the day prior to and approximately 1 month following surgery. All patients underwent either a classic Whipple's procedure or a pylorus preserving resection for removal of ductal adenocarcinoma of the head of pancreas. Patients with ampullary, periampullary and duodenal carcinoma were excluded from study as well as pancreatic neuroendocrine tumours. Patients presenting either with cholangitis or other clinical evidence of infection, especially where the bile culture was positive, were also excluded from study.

All patients were treated in the upper GI surgical unit at Glasgow Royal Infirmary and survived at least 30 days following surgery. No patient underwent chemotherapy. The study was approved by the local ethical committee.

### Methods

Tumours were classified by type, along with pathological staging criteria (size, nodal status, perineural and vascular invasion, and tumour differentiation). Tumour stage was according to the International Union Against Cancer Criteria (IUCC). Tumour size was taken as the largest tumour diameter measured by the pathologist.

Routine laboratory measurements of albumin and bilirubin were carried out. C-reactive protein was measured by Fluorescence Polarisation Immunoassay using an Abbott TDX analyser and Abbott reagents (Abbott Laboratories, Abbott Park, IL, USA). The coefficient of variation for this method, over the range of measurement, was less than 5% as established by routine quality control procedures. The limit of detection of the assay is a C-reactive protein concentration of less than 5 mg l^−1^ with the upper limit of normal values being ⩽10 mg l^−1^. Based on previous work (O’Gorman *et al*, 2000), a C-reactive protein concentration of greater than 10 mg l^−1^ was considered to indicate the presence of a systemic inflammatory response.

### Statistics

Comparisons between groups of patients were carried out using contingency table analysis (*X*^2^) as appropriate. Survival analysis was performed using the Cox's proportional-hazards model. Deaths up to the end of March 2004 were included in the analysis. Multivariate survival analysis was performed using a stepwise backward procedure to derive a final model of the variables that had a significant independent relationship with survival. To remove a variable from the model, the corresponding *P*-value had to be greater than 0.10. Analysis was performed using SPSS software (SPSS Inc., Chicago, IL, USA).

## RESULTS

Baseline characteristics of the patients (*n*=65) studied are shown in Table 1. The majority of patients were under 65 years with similar numbers of male and female patients. The majority of patients had stage III disease (IUCC), positive circumferential margin involvement (R_1_), tumour size greater than 25 mm with perineural and lymph node invasion. The tumour was well differentiated in 12%, moderate in 55% and poor in the 32%.

In all, 33 patients (51%) had evidence of a systemic inflammatory response preoperatively, that is, circulating C-reactive protein concentration greater than 10 mg l^−1^. During the follow-up period, 60 (92%) patients died. The median survival time was 13.4 months.

On univariate analysis, sex (*P*<0.05), tumour size (*P*<0.05), vascular invasion (*P*<0.001), preoperative (*P*<0.001) and postoperative (*P*<0.001) C-reactive protein concentrations were significantly associated with survival (Table 1). On multivariate analysis of these significant variables, tumour size (hazard ratio (HR) 2.10, 95% confidence interval (CI) 1.20–3.68, *P*=0.009), vascular invasion (HR 2.58, 95% CI 1.48–4.50, *P*<0.001) and postoperative C-reactive protein (HR 2.00, 95% CI 1.14–3.52, *P*=0.015) retained independent significance. The patient group with no evidence of a postoperative systemic inflammatory response (C-reactive protein ⩽10 mg l^−1^) had a median survival of 21.5 months compared with 8.4 months in the elevated systemic inflammatory response group (*P*<0.001).

The relationship between the presence of an elevated preoperative C-reactive protein concentration and tumour characteristics are shown in Table 2. There was no significant difference in the stage, presence of positive resection margins, lymph node metastases or perineural invasion between the inflammatory and noninflammatory groups. There were a greater number of males (*P*<0.05) and tumours were larger (*P*<0.05), had more vascular invasion (*P*<0.05) and had poorer differentiation (*P*<0.05) in the elevated C-reactive protein group.

## DISCUSSION

Surgical resection remains the only prospect for long-term survival in patients with ductal adenocarcinoma of the pancreas. Currently, in patients undergoing surgery, prognostic factors are all based on the pathological findings from the resected tumour. However, this means that the assessment of prognosis occurs following a major operation with significant morbidity and mortality. Therefore, it is of interest that in the present study an elevated circulating concentration of C-reactive protein (>10 mg l^−1^), measured preoperatively, was associated with poor survival and was associated with pathological criteria indicative of poor outcome.

It has been previously shown that, in patients with primary operable colorectal cancer, approximately one-third of patients had an elevated circulating concentration of C-reactive protein preoperatively and that these patients had a significantly poorer outcome (McMillan *et al*, 2003). It was of interest that, in the present study, the proportion of patients with an elevated preoperative C-reactive protein concentration was approximately half and that these patients also had a poorer outcome. It may be that because C-reactive protein concentration is independent of tumour stage, it might form the basis of a new prognostic score that reflects not only the tumour response but also that of the host. Indeed, this approach has recently been used to improve the prediction of outcome in patients who underwent potentially curative resection for oesophageal and colorectal cancer (Ikeda *et al*, 2003; Canna *et al*, 2004).

In the present study, an elevated C-reactive protein concentration, measured either pre- or postoperatively, was associated with more than a halving of survival. When, in multivariate survival analysis, an elevated preoperative C-reactive protein was compared with the postoperative (1 month) value, an elevated postoperative C-reactive protein better predicted poor survival independent of pathological criteria. Although from the present results an elevated postoperative C-reactive concentration is a better predictor of survival than an elevated preoperative value, in a clinical context, the measurement of pre- and postoperative C-reactive protein concentrations are both likely to be of value. The preoperative measurement, since it may allow planning of adjuvant treatment, and the postoperative measurement, since it may be useful in the monitoring of patients who have undergone resection for ductal adenocarcinoma of the head of the pancreas.

This is a retrospective study and requires verification in larger prospective cohorts. However, if an elevated C-reactive protein concentration is shown to predict a poorer prognosis, it may be the case that patients with potentially resectable ductal adenocarcinoma of the head of the pancreas, yet a high inflammatory profile preoperatively, should not undergo surgery. Alternatively, modulation of the systemic inflammatory response may be a useful approach in these patients in the postoperative period.

In summary, the results of the present study indicate that, in patients who have undergone potentially curative resection for ductal adenocarcinoma of the head of pancreas, the presence of a systemic inflammatory response predicts poor outcome.

## Figures and Tables

**Table 1:** Characteristics of patients who underwent potentially curative resection for ductal adenocarcinoma of the head of the pancreas: univariate survival analysis

	**Patients (*n*=65)**	**HR (95% CI)**	***P*-value**
Age (<65/>65 years)	37/28	0.66 (0.39–1.12)	0.127
Sex (f/m)	33/32	1.78 (1.06–2.99)	0.031
Stage (I/II/III)	17/2/46	1.13 (0.83–1.53)	0.449
			
Resection margin R0/R1	19/46	1.64 (0.92–2.91)	0.094
Tumour size (⩽25/>25 mm)	27/38	1.99 (1.14–3.45)	0.015
Lymph node invasion (−/+)	20/45	1.25 (0.71–2.22)	0.444
Perineural invasion (−/+)	6/59	1.01 (0.44–2.37)	0.973
Vascular invasion (−/+)	39/26	2.75 (1.60–4.71)	<0.001
Tumour differentiation (well/moderate/poor)	8/36/21	1.46 (0.97–2.20)	0.072
			
Albumin (⩾35/<35 g l^−1^)	40/25	1.00 (0.59–1.71)	0.987
Bilirubin (⩽22/>22 *μ*mol l^−1^)	12/53	1.12 (0.56–2.23)	0.750
Biliary stent (no/yes)	32/33	1.06 (0.63–1.76)	0.836
Preoperative			
C-reactive protein (⩽10/>10 mg l^−1^)	32/33	2.56 (1.51–4.36)	<0.001
Postoperative			
C-reactive protein (⩽10/>10 mg l^−1^)	33/32	2.50 (1.46–4.29)	<0.001

CI=confidence interval; HR=hazard ratio.

**Table 2:** The relationship between the presence of a preoperative systemic inflammatory response and tumour characteristics of ductal adenocarcinoma of the head of the pancreas

	**C-reactive protein ⩽10 mg l^−1^ (*n*=32)**	**C-reactive protein>10 mg l^−1^ (*n*=33)**	***P*-value**
Age ⩽65/>65 years)	15/17	22/11	0.107
Sex (f/m)	21/11	12/21	0.018
Stage (I/II/III)	9/2/21	8/0/25	0.302
			
Resection margin R0/R1	11/21	8/25	0.369
Tumour size (⩽25/>25 mm)	18/14	9/24	0.018
Lymph node invasion (−/+)	12/20	8/25	0.247
Perineural invasion (−/+)	4/28	2/31	0.370
Vascular invasion (−/+)	24/8	15/18	0.015
Tumour differentiation (well/moderate/poor)	6/21/5	2/15/16	0.013
			
Albumin (⩾35/<35 g l^−1^)	23/9	17/16	0.092
Bilirubin (⩽22/>22)	26/6	27/6	0.953
Biliary stent (no/yes)	18/14	14/19	0.265
Postoperative			
C-reactive protein (⩽10/>10 mg l^−1^)	17/15	10/23	0.062
			
Survival (months)^a^	18.2 (14.9–21.4)	8.3 (6.6–10.0)	<0.001

a Median (95% confidence interval).
